# Clinical Lecture on Facial Palsy

**Published:** 1879-10

**Authors:** H. C. Wood


### CLINICAL LECTURE ON FACIAL PALSY.

BY PROF. H. C. WOOD, JR., M.D.

Gentlemen—I bring before you a case which offers a great deal of interest in regard to the diagnosis of its cause.

This young woman has been out of health for three months. Her first symptoms were severe headache, referred chiefly to the region of the mastoid process of the temporal bone of the right side, and accompanied by occasional spells of giddiness. About six weeks ago she noticed for the first time that she would stagger, or even fall, on attempting to walk. To-day she complains of weakness in her limbs and inability to direct their movements. Her right ear cannot appreciate the tick of the watch even when it is applied close against the head; she has lost the sense of taste on the tip of the tongue of the side affected. She has a tumor under the lower angle of the left scapula which I believe to be a neuroma, on account of its exquisite tenderness on pressure.

By looking at her face you can at once detect that her right side is paralyzed; her mouth is drawn a little towards the sound side; if I told her to blow, her right cheek would bulge out, and, in eating, food accumulates between the gum and the buccinator muscle. If I tell her to close her eyes, her right eye remains open, and her forehead is utterly expressionless.

What is the origin of this palsy? Have we to deal with a paralysis of peripheral or of centric origin? I believe it to be peripheral, because we have an entire and uniform paralysis of this side of the face. If it were of centric origin we should have scattered face-areas affected, because the portio dura arises by several disseminated centres, and some fibres would probably escape the lesion. It is by reason of this manifold origin of the facial nerve that complete facial palsy is never centric. It is true that there are in this patient certain symptoms—as the staggering and giddiness—which would suggest a centric origin of the palsy, but these symptoms can be explained otherwise.

The loss of hearing is nervous in its character, and not due to a disease of the ear membrane, for we know that if the vibrations of a tuning-fork are not appreciated when we place it upon the side of the head it means either that the nerve of that side is absent or has been incapacitated by disease to perform its function.

The loss of taste can be accounted for by remembering that in facial palsy, when the lesion is so far back as to affect the nerve-trunk before the chorda tympani is given off, through paralysis of that nerve the secretion of saliva is interfered with and the function of taste is also upon the anterior portion of the tongue on that side. The fact that the loss of taste in this woman is on the same side as the palsy is a very strong indication that the paralysis is of peripheral origin, for a centric lesion destroying taste and motion on the same side in the localized manner here present is an almost unheard-of rarity, if indeed it be at all possible.

The symptoms that would suggest a centric origin are staggering, giddiness, and inco-ordination of movements; but these may be due to a local peripheral lesion. In the internal ear we have the organs known as the semicircular canals, which probably are not connected simply with the function of hearing, for experimental as well as clinical evidence shows that they are largely engaged in maintaining the equilibrium between the individual and the external world. Thus, if in a bird we destroy these canals, we will see it turning around and around, always toward the injured side, or, in other words, performing what are called circus movements. In frogs wounds of the ear produce a similar loss of power on the injured side. In Meniere's disease, you know, apoplexy into the labyrinth is at once followed by staggering, giddiness, etc. Two or three years ago I witnessed a case in which, the man having been shot in the face, a bullet was lodged near the foramen through which the portio dura and the portio mollis enter together; thus pressing on these nerves, it affected profoundly their functional powers, producing phenomena precisely parallel to those which are seen in the animal whose semicircular canals have been injured.

Most probably in this young woman we have a similar condition; not that the semicircular canals are destroyed, but possibly the function of the portio mollis is in some way interfered with by pressure. Another proof that the paralysis is a peripheral one is that the muscles answer much more readily to the continued current than to the galvano-faradic one.

Considering it proven that the paralysis is of peripheral origin, what is the nature of the lesion? It may be rheumatic, or due to disease of the petrous portion of the temporal bone, or to thickening of the membrane lining the aqueduct of Fallopius, or to an injury, or to a chronic tuberculous inflammation of the brain, or, lastly, to pressure by a tumor. We can at once exclude number four, for there is no history of a blow having been received; so can we also exclude number five, for the general appearance and general health of the patient are good, and there is no tenderness or any other local indication of disease of this bone. If the palsy were rheumatic it would have come on suddenly; and in this case the disease has been progressive. It is not due to disease of the petrous portion of the temporal bone, for we have no local tenderness, no signs of suppuration or history of long-standing disease of the ear.

We cannot entirely exclude the thickening of the membrane lining the aqueductal Fallopii, but it is doubtful whether this thickening would be such as completely to obliterate the canal and paralyze the nerves. Moreover, there is no apparent cause for this thickening, and the history of the case is altogether too acute for such a supposition, although not acute enough for the theory of a rheumatic attack. The most plausible explanation of the present phenomena is, according to my views, pressure exerted by a growth, with which it may be, co-operates with some thickening of the membrane lining the aqueduct; the character of this foreign body I believe to be specific, although there is no absolute proof of such origin.

*Treatment.*—We will give our patient the benefit of the doubt, and will place her on large doses of the iodide of potassium, combined with the bichloride of mercury. If the trouble were rheumatic, we would place her on the salicylates, although in our hands they have proven of much less service in the chronic or subacute forms of rheumatism than in the acute forms of the disease. If this trouble is rheumatic in its origin it will be benefitted by doses of iodide of potassium, smaller, however, than those given for syphilis.

The use of electricity is advisable, not to cure the disease, but to keep up the tone and proper nutrition of the muscles.

We have not used any blisters behind the ear. The patient is improving under the specific treatment; the tumor at the angle of the scapula is less tender on pressure.

[Under specific and local treatment the patient continued to improve, and was subsequently shown to the class almost recovered.—*Philadelphia Medical News*.

